# The Association Between Patient Visit Demographics and Opioid Analgesic Received in the Emergency Department

**DOI:** 10.7759/cureus.5678

**Published:** 2019-09-17

**Authors:** Logan J Richards, Nicholas J Hopkins, Nicholas A Colwell, Abe E Sahmoun, James R Beal

**Affiliations:** 1 Miscellaneous, University of North Dakota School of Medicine and Health Sciences, Bismarck, USA; 2 Miscellaneous, University of North Dakota School of Medicine, Grand Forks, USA; 3 Family & Community Medicine, University of North Dakota School of Medicine, Grand Forks, USA; 4 Internal Medicine, University of North Dakota School of Medicine and Health Sciences, Fargo, USA

**Keywords:** emergency department, epidemiology, opiate, opioid, bias, demographics, race, age, prescribing

## Abstract

Introduction: Minimal research has been conducted on the prescribing patterns of emergency room physicians. The opioid epidemic is a well-known public health crisis and increased knowledge of providers’ tendencies to prescribe opioids over other analgesia may help to update guidelines, improve patient safety, and lower the amount of opioid diversion and death from overdose. The purpose of this study was to determine the association between patient visit demographics and prescribed opiate analgesics.

Methods: We conducted a retrospective study analyzing adult patient visits that were seen in the emergency setting for acute pain including chest pain, back pain, abdominal pain, headache, face/tooth/ear, or musculoskeletal pain, utilizing the 2011-2016 National Hospital Ambulatory Medical Care Survey Emergency Department Patient Record dataset. We analyzed the relationship between various patient visit characteristics and whether opiate analgesics were given or prescribed.

Results: Our study included 73,983 visits for pain, representing an estimated 407 million weighted visits over the study period. We found that those who received opiates were more likely to be female, 62.9% vs. 60.2% and more likely to be white, 74.2% vs. 71.3 %. Furthermore, visits that received opiates were more likely to be younger, have private insurance, and be in increased pain (all P-values = 0.000).

Conclusion: We found that certain patient visit characteristics - including being female, white, younger, and private insurance - were given opiates more in the emergency department. Females have been found to report more pain, the elderly have special considerations regarding pain medications (including the risk of delirium and drug-drug interactions), while insurance status may be confounded by age (Medicare being a large portion of government insurance). However, explanations for differences in prescription rates by race could not be easily discerned.

## Introduction

The opioid epidemic is a well-known public health crisis. Economically, the nonmedical use of prescription opioids in 2007 was estimated at $55.7-$25.6 billion due to workplace costs, $25 billion due to healthcare costs, and $5.1 billion due to criminal justice costs [1]. From a humanitarian perspective, more than 130 individuals are killed each day in the United States due to opioid drug overdose [2]. Emergency departments (EDs) are thought to be a major contributor to the opioid epidemic [3]. EDs are responsible for the treatment of opioid drug overdose as well as a common source of opioid prescriptions [4]. The most common complaint of a patient visiting the ED is pain, and emergency medicine physicians often treat pain with opioid pain relievers [5]. Nearly two-thirds of patients use the ED for acute pain or exacerbations of chronic pain [6] and one-third of all ED patients will be given an opioid during their visit or be prescribed an opioid at discharge [7]. Due to the large quantity of opioid administration, lack of provider continuity, pressure in the ED to turnover patients, and limitations in available patient information in emergency settings, EDs are susceptible to drug diversion [3]. For this reason, there has been an emphasis placed on opioid pain reliever prescribing guidelines and prescription drug monitoring programs to potentially decrease diversion and overdose rates [6]. Along with these interventions, there has been an increase in the study of prescribing patterns of emergency department physicians [6]. Increased knowledge of providers’ tendencies to prescribe opioid pain relievers over another form of analgesic would help us to update guidelines and potentially lower the amount of opioid diversion and deaths due to opioid overdose. Additionally, it may uncover potential bias in opiate prescription patterns regarding non-modifiable patient characteristics. The purpose of this study was to determine the association between patient demographics and if opiate analgesics were given or prescribed during a visit to the emergency department.

## Materials and methods

We conducted a retrospective study analyzing adult patients, 18 years or older, that were seen in the emergency setting with a reason for the visit of acute pain using the 2011-2016 National Hospital Ambulatory Medical Care Survey (NHAMCS) Emergency Department patient record dataset. The NHAMCS-ED is an annual multistage probability sample of visits made to United States emergency departments, conducted by the Center of Disease Control National Center for Health Statistics. Acute pain included chest pain, back pain, abdominal pain, headache, or other pain. We excluded patient visits who presented with poisoning, injury, or overt trauma as well as pregnant patients. The Multum Lexicon Plus system was used to classify drugs and identify opioids which included codeine, fentanyl, hydrocodone, hydromorphone, meperidine, morphine, oxycodone, tramadol, and any of these drugs used as a single agent or in combination with other medications. We evaluated whether patients were given or prescribed opioids, single and combinations, or nonsteroidal anti-inflammatory drug (NSAID)/acetaminophen analgesia during their visit. Independent variables used during this study included gender, race, age, pain scale, insurance status, whether the patient was seen in the emergency department within the last 72 hours prior to present visit, whether the visit took place in a Metropolitan-Statistical-Area (MSA) or non-MSA, and geographical census region that the visit took place in (Northeast, Midwest, South, or West).

The data was analyzed using Statistical Package for Social Sciences (SPSS) Complex Samples 25.0 in a manner that accounted for the NHAMCS complex sample survey design. We analyzed the data using summary statistics and bivariate comparisons such as Chi-square tests. All significant tests were two-sided, and we used a p-value of less than .05 for significance. Institutional Review Board approval was obtained through the University of North Dakota.

## Results

Our study included 73,983 visits for pain, representing an estimated 407 million weighted visits over the study period. During which, 61% of visits were female patients while patients had a mean age of 45.9±0.2 years. Opiates were given at approximately 30.2% of visits. We found that those who received opiates were significantly more likely to be female (P = 0.000), 62.9% to 60.2% [Table 1]. Additionally, those who received opiates were significantly more likely to be in the youngest age group (18-44), 54.0% vs. 49.3%; and less likely to be in the oldest age group (65 and older), 13.9% vs. 23.9% (P = 0.000). Race was also found to be a significant characteristic, as those who received opiates were significantly more likely to be white, 74.2% vs. 71.3 % (P = 0.000). In contrast, those who received opiates were significantly less likely to be black, 23.0% vs 25.3%, [Table 1]. Additionally, visits that received opiates were significantly more likely to have private insurance and less likely to have government insurance than visits not receiving opiates, 32.6% vs 29.3% and 47.3% vs. 53.4%, (P = 0.000)[Table 1]. Furthermore, those who received opiates were significantly more likely to: be in increased pain (pain scale 7-10, 71.1% vs 34.2%), have been seen in the ED in the last 72 hours (5.0% vs 4.3%), live in a Metropolitan Statistical Area (84.7% vs 82.6%), and live in the South or West regions of the United States (40.7% vs 37% and 23.5% vs 20.6%, respectively), [Table 1]. Furthermore, about two-thirds (66.4%) of those who received opiates had additionally received NSAIDs or acetaminophen during their visit.

**Table 1:** Characteristics of patient visits by share of total population and share of opiate receiver/non-receiver population *N = 407,612,058, UN = 73,983, 100% **N = 123,125,891, UN = 21,938, 30.2% ***N = 284,486,166, UN = 52,045, 69.8% -UN=Unweighted number of visits -%'s are estimated percentages Abbreviations: ED: Emergency Department; MSA: Metropolitan Statistical Area; NSAIDs: Nonsteroidal anti-inflammatory drugs

Characteristics	Total* (%)	Opiates** (%)	No Opiates*** (%)	p-value
Gender:				0.000
Female	61.0	62.9	60.2	
Male	39.0	37.1	39.8	
Race:				0.000
White	72.2	74.2	71.3	
Black	24.6	23.0	25.3	
Other	3.2	2.7	3.4	
Age:				0.000
18-44 years	50.7	54.0	49.3	
45-64 years	28.4	32.1	26.8	
65 years and over	20.9	13.9	23.9	
Pain Scale:				0.000
0	25.3	5.7	34.8	
1-3	8.1	4.8	9.7	
4-6	20.4	19.4	21.3	
7-10	46.2	71.1	34.2	
Insurance Status:				0.000
Private	30.3	32.6	29.3	
Government	51.6	47.4	53.4	
Self	14.3	16.0	13.6	
Other	3.8	4.0	3.7	
Seen in ED in last 72 hours:	4.5	5.0	4.3	0.011
MSA:	83.3	84.7	82.6	0.023
Non-MSA:	16.7	15.3	17.4	
Geographical Region:				0.000
Northeast	17.6	12.9	19.6	
Midwest	22.8	22.9	22.8	
South	38.1	40.7	37.0	
West	21.5	23.5	20.6	
NSAIDs and Acetaminophen:				0.000
Yes	73.0	66.4	75.9	
No	23.0	33.6	24.1	

## Discussion

We found that certain patient visit characteristics, including being white, female, and younger, were more likely to be given or prescribed opiates in the emergency department. Visits that were given or prescribed opiates were more likely to be white and less likely to be black or other races. Pletcher et al. reported similar significant differences in opioid prescribing by race/ethnicity in US emergency departments, finding that white patients were more likely to receive opioids than black patients. It was further reported that this prescribing differential became more evident as patient-reported pain severity increased, with adjustment for severity not substantially affecting the differences. The differential persisted throughout all types of pain visits [8]. Moreover, a review article described numerous emergency physicians pain management biases, including ethnic/racial bias, gender bias, and age bias. They described that female patients have been found to report more pain and have been perceived by providers to be in more pain while receiving more pain medication as well as stronger medications. Similar findings were present with regards to age, with an elderly patient receiving fewer medications than their younger counterparts [9]. Though the physiologic differences and rationale behind these biases were not thoroughly discussed, these results are concurrent with our findings that opioid receivers were more likely to be female and in the younger age group.

According to the American Geriatrics Society Beers Criteria, which describes potentially unsuitable prescription medications for the elderly, only meperidine was included in our protocol. No other opiate was listed as inappropriate for older adults. However, physicians prescribing opioid medications to the elderly have special considerations that do not have to be thought of when giving opioids to younger populations. Older adults are at an increased risk of developing delirium, increased risk of a drug-drug interaction, and increased risk of a drug-disease interaction when taking opioid pain relievers [10]. Additionally, the use of opioid medication puts adults over the age of 65 at an increased risk for fracture or soft tissue injury [11]. These potential adverse effects of opiates in older adults could explain the decreased utilization of opioids as pain relievers in adults over the age of 65. The adverse effects of opioids in the elderly can also, partially, explain the relationship between government insurance status and the likelihood to be prescribed opioids. Medicare is a large government insurance plan and is mainly made up of individuals over the age of 65. Therefore, if physicians are not prescribing opioids to older individuals based on the aforementioned adverse effects, this will also influence the results of the government insurance status and administration of opioid pain relievers due to Medicare making up a large portion of government insurance plans.

Several additional reasons that influenced patients receiving opioids during their ED visit have been previously addressed. Some physicians have admitted to giving opioid analgesic to patients with the intent of improving patient satisfaction results [12]. Others have used the prescription of opioids to expedite discharge [12]. Ultimately, prescribing differences between individual providers will always be a factor in our society’s usage of opioids [13]. If it is to be believed that all it takes is one instance of opioid ingestion to become addicted, prescribers using any other criteria other than the legitimate need for opioids is a huge concern. It remains unclear as to how big a role these factors have played/continue to play in how our society utilizes opioid resources, but it does add to the conversation in terms of the opioid epidemic.

Strengths of our study included a national dataset with a large sample size. However, having a large sample size may result in small differences being found as statistically significant without necessarily being clinically relevant. Limitations included pain being self-reported by the patient, making this variable subjective.

## Conclusions

We found that those who received opiates in the ED were more likely to be female, white, younger, and have private insurance. Differences in opioid prescription rates due to patient gender, age, or insurance status may be due to logical reasons as females have been found to report more pain, the elderly have special considerations regarding pain medications (including risk of delirium and drug-drug interactions), while insurance status may be confounded by age (Medicare being a large portion of government insurance). However, explanations for differences in prescription rates by race, could not be easily discerned. Future research in this area will help elucidate and more clearly quantify the presence of bias in opiate prescription patterns regarding non-modifiable patient characteristics.
